# Motor neuron involvement expands the neuropathological phenotype of late‐onset ataxia in *RFC1* mutation (CANVAS)

**DOI:** 10.1111/bpa.13051

**Published:** 2022-01-09

**Authors:** David Reyes‐Leiva, Iban Aldecoa, Ellen Gelpi, Ricard Rojas‐García

**Affiliations:** ^1^ Neuromuscular Diseases Unit Department of Neurology Hospital de la Santa Creu i Sant Pau IIB Sant Pau Universitat Autonoma de Barcelona Barcelona Spain; ^2^ Neurological Tissue Bank of the Biobank‐Hospital Clinic‐IDIBAPS Barcelona Spain; ^3^ Department of Pathology Biomedical Diagnostic Centre (CDB) Hospital Clinic – University of Barcelona Barcelona Spain; ^4^ Division of Neuropathology and Neurochemistry Department of Neurology Medical University of Vienna Wien Austria

**Keywords:** ataxia, CANVAS, RFC1 neuropathology, vestibular

## Abstract

Neuropathological features in brainstem, cerebellum and spinal cord. In addition to cerebellar, vestibullar nuclei and spinal cord posterior columns involvement, a moderate reduction of motor neurons in hypoglossal nucleus and anterior horn of the thoracic spinal cord was present.

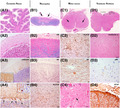

## CONFLICT OF INTEREST

David Reyes‐Leiva, Iban Aldecoa, Ellen Gelpi and Ricardo Rojas‐Garcia report no disclosure or conflict of interest.

Recently, a RFC1 repeat expansion has been reported in “cerebellar ataxia, neuropathy and vestibular areflexia syndrome [1]. In this article, the authors report the clinical description of a large cohort of patients affected with cerebellar ataxia, neuropathy and vestibular areflexia syndrome (CANVAS) that share a common mutation in the *replication factor complex subunit 1* (*RFC1*) gene. In addition to the clinical classical presentation, further reports have highlighted the huge range of neurological signs and symptoms in those patients during disease course [1, 2, 3, 4].

As neuropathological descriptions are scarce [5] and some less frequent features have been reported since its genetic basis is known, we aimed to describe the clinical and neuropathological findings in a patient who presented late‐onset cerebellar ataxia with sensory neuronopathy and RFC1 mutation who additionally had lower motor neuron involvement at the neuropathological examination.

The patient was first seen in our clinic in 2003 at the age of 73 for distal dysesthesia for several months. He had no positive family history for any neurological disorder. At that moment he did not complain of cough or dysautonomic symptoms. Neurological examination revealed ataxic gait, socks and gloves hypoesthesia and hypopalesthesia, and brisk tendon reflexes. No spasticity and no Babinski sign were evidenced. Ocular motricity was preserved, but he had horizontal nystagmus in lateral gaze to both sides. Mild bilateral ataxia was noticed in the nose‐finger manoeuvre. Vestibular testing was not performed. The electrophysiological study performed at the first visit revealed no electrical response in any tested sensory nerve, of the upper and lower extremities. Motor nerve conductions and needle electromyography at that moment were both normal. Brain MRI revealed a pan‐cerebellar atrophy without any additional radiological findings.

Blood tests including most common causes of sensory polyneuropathy were normal. Genetic analysis for hereditary ataxias including Friedreich ataxia, SCA gene panel and DRLPA did not identify causative mutations. Therefore, the patient was diagnosed with a sensory neuronopathy with cerebellar ataxia of unknown aetiology.

During the following years patient's ataxia worsened until he got wheelchair bound. He progressively developed severe dysphagia leading to aspiration pneumonia, respiratory distress and finally to death at the age of 88 years, 15 years from symptom onset. The patient had consented to brain donation for research purposes at the IDIBAPS Brain Bank. A genetic testing from archival peripheral blood focused on the analysis of the *RFC1* intron 2 based on Repeated Primed PCR for the pathologic allele (AAGGG)exp was conducted posthumously, as mutations in *RFC1* are currently considered to be the main cause of late‐onset ataxia with neuronopathy as part of the CANVAS spectrum [1, 2, 3, 4]. We identified a homozygous AAGGG intronic expansion in intron 2, compatible with the diagnosis of CANVAS.

**FIGURE 1:**
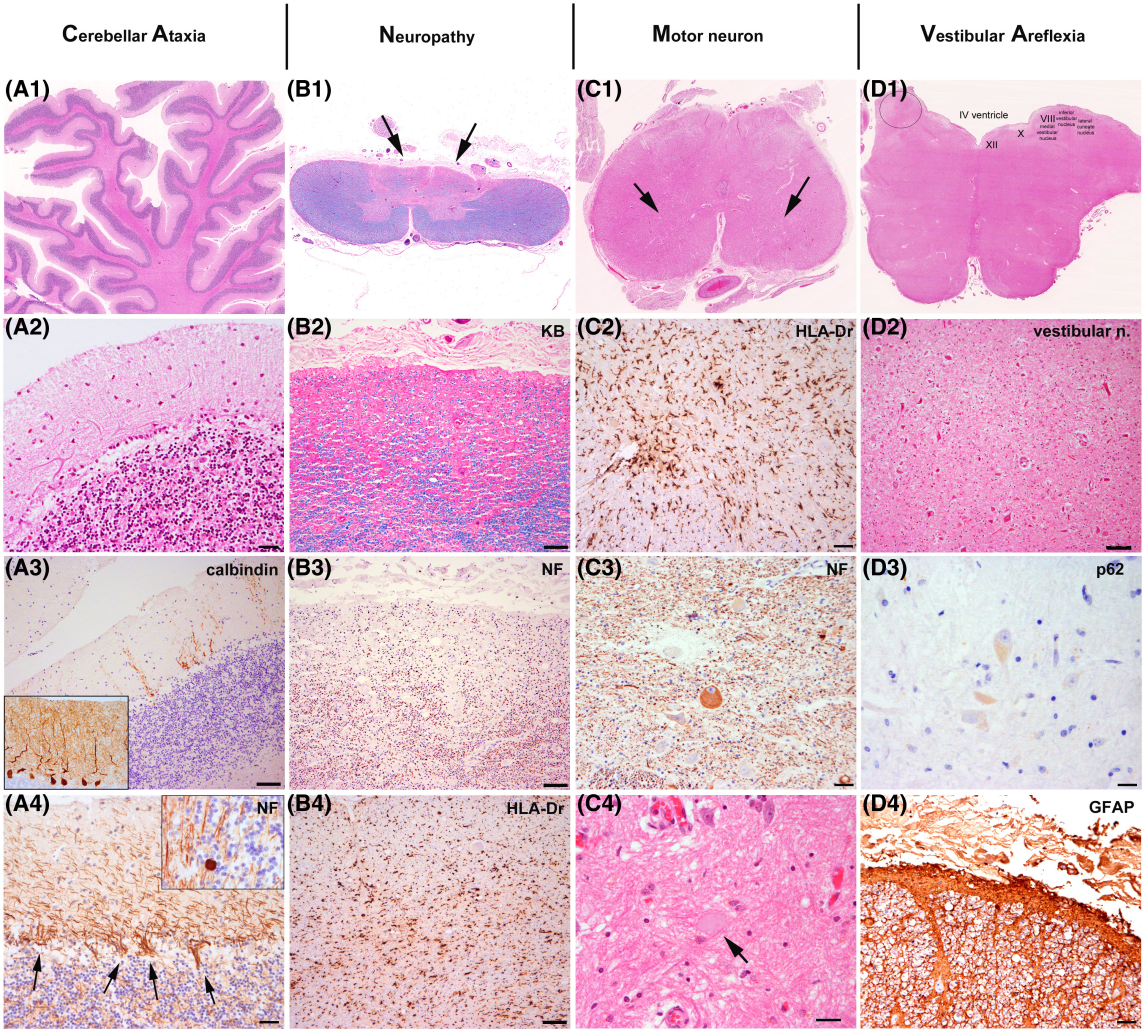
Characteristic neuropathological features of CANVAS. (A1–A4) representing the cerebellar degeneration. (B1–B4) representing the degeneration of the posterior columns. (C1–C4) representing the lower motor neuron involvement. (D1–D4) representing the degeneration of the vestibular nuclei. (A1) Atrophy of the cerebellar folia (haematoxylin–eosin stain). (A2) Loss of Purkinje cells and proliferation of Bergmann glia. (A3) Calbindin immunohistochemistry depicts the prominent loss of Purkinje cells and of their apical arborization. (A4) Immunohistochemistry for neurofilaments reveals abundant ‘empty baskets’ (arrows) and axonal spheroids of the degenerating Purkinje cells within the granule cell layer (torpedoes) (inset). (B1) Cross section through the thoracic spinal cord shows severe flattening of the posterior columns (Klüver–Barrera stain: luxol fast blue for myelin sheaths and nuclear read for cells). (B2) higher magnification shows prominent loss of myelin sheaths (Klüver–Barrera stain) and of axonal profiles (B3: immunohistochemistry for phosphorylated neurofilaments) with an increase in microglia/macrophages (B4: immunohistochemistry for HLA‐DR). (C1) Cross section of the lumbosacral spinal cord reveals a reduction of motor neurons of the anterior horn. This is associated with an increase in microglia/macrophagic profiles (C2: immunohistochemistry for HLA‐DR), accumulation of phosphorylated neurofilaments in some chromatolytic neurons (C3: immunohistochemistry for neurofilaments) and formation of axonal spheroids (C4: haematoxylin–eosin; arrow points to spheroid). (D1) At the level of the medulla oblongata there is a prominent degeneration of the vestibular nuclei with neuronal loss and marked reactive gliosis (D2: higher magnification). (D3) No intranuclear or cytoplasmic inclusion bodies are detected with anti‐p62 antibody in the affected brainstem, spinal cord or cerebellar regions. (D4) Immunohistochemistry for glial fibrillary acidic protein (GFAP) shows a band‐like subpial fibrillary gliosis around the spinal cord. Scale bars: A2, A4, C3, D4: 20 µm; A3, B2, B3, B4, C2, D2: 50 µm; C4: 21 µm; D3: 10 µm

The neuropathological study is shown in Figure 1. The most prominent post‐mortem neuropathological features were observed in the brainstem, cerebellum and spinal cord. We observed a moderate bilateral degeneration of the vestibular nuclei at the level of the pons and medulla oblongata with neuronal loss, astrogliosis and microglial activation (Figure 1D1–D3). The pontine nuclei of the ventral pons were preserved. There was a moderate reduction of motor neurons of the hypoglossal nucleus and especially of the anterior horn of the spinal cord (Figure 1C1–C4), particularly at the thoracic level. Chromatolytic neurons and axonal spheroids were also identified (Figure 1C4). No signs of cortico‐spinal tract degeneration were observed. In the spinal cord, there was a severe degeneration of the posterior columns with marked flattening of the surface (Figure 1B1–B4). A chronic band‐like subpial fibrillary gliosis was observed along the brainstem and spinal cord (Figure 1D4). The cerebellar vermis showed an extensive loss of Purkinje cells while it was segmental in the hemispheres (Figure 1A1–A3). This was associated with Bergmann gliosis (Figure 1A2), diffuse white matter rarefaction and a diffuse gliosis of the dentate nucleus. Also the interconnected inferior olivary nuclei were moderately gliotic. The granule cell layer was comparatively well preserved. Besides moderate concomitant small vessel disease and Alzheimer's disease neuropathological changes of intermediate severity (A2, B2 and C2) [6] we could not identify obvious intranuclear or intracytoplasmic neuronal or glial inclusions in the affected areas; also, immunohistochemical stains for ubiquitin, p62, tau, alpha‐synuclein, neurofilaments, ßA4, pTDP43, FUS, C9RANT and polyQ remained negative in the brainstem, spinal cord and cerebellum. Unfortunately, dorsal root ganglia were not available for analysis.

The neuropathological features were consistent with those described in CANVAS [5] allowing for the categorization as ‘pathologically definite CANVAS’ according to suggested criteria [7]. While no specific vestibular testing was performed in our patient during life because cerebellar symptoms were the primary complaint, neuropathological examination revealed a moderate degeneration of the vestibular nuclei. Moreover, and although the dorsal root ganglion could not be assessed neuropathologically, the electrophysiological study was compatible with a dorsal root ganglion neuronopathy. These findings, along with the additional neuropathological features and the genetic results support the diagnosis of CANVAS.

The moderate involvement of motor neurons of the brainstem and the spinal cord, which was more than expected for mere ageing, has, to our knowledge, not been described so far. Needle electromyography examination was only performed early in the diagnostic work‐up and was not repeated because of lack of limb weakness or atrophy during the disease course. Bulbar weakness was assumed to be part of the disease process. However, our findings suggest that these symptoms could be also a consequence of motor neuron involvement at the brainstem level. The loss of motor neurons of the hypoglossal nucleus and spinal cord may play a role in the development of motor manifestations such as dysarthria and dysphagia, a frequent feature that CANVAS patients develop during disease course [1]. Therefore, lower motor neuron involvement may be part of the clinical and neuropathological spectrum of CANVAS. We believe it should not represent a strict exclusion criteria and should be taken into account in patients with suspected CANVAS to precisely define the whole spectrum of manifestations of the disease.

Many neurodegenerative diseases are considered protein misfolding disorders and the abnormal protein aggregates may be identified in brain tissue by appropriate specific antibodies, including some genetic diseases with intronic expansions (e.g. C9orf72). However, we could not identify bona fide ubiquitinated/p62+ inclusions in affected or unaffected brain regions, neither in neurons nor in glial cells that could represent a morphological clue for an underlying proteinopathy in CANVAS.

In sum, this case expands neuropathological features of a genetically confirmed CANVAS underscoring its multiple system character, including the potential involvement of lower motor neurons. Future studies on the expansion and its product will help to elucidate mechanisms of neuronal damage in these particularly vulnerable brain regions.

## Data Availability

The data that support the findings of this study are available on request from the corresponding author.

## References

[1] Cortese A , Tozza S , Yau WY , Rossi S , Beecroft SJ , Jaunmuktane Z , et al. Cerebellar ataxia, neuropathy, vestibular areflexia syndrome due to RFC1 repeat expansion. Brain. 2020;143(2):480–90. 32040566 10.1093/brain/awz418 PMC7009469

[2] Cortese A , Simone R , Sullivan R , Vandrovcova J , Tariq H , Yau WY , et al. Biallelic expansion of an intronic repeat in RFC1 is a common cause of late‐onset ataxia. Nat Genet. 2019;51(4):649–58. 30926972 10.1038/s41588-019-0372-4 PMC6709527

[3] Cortese A , Reilly MM , Houlden H . *RFC1* CANVAS/spectrum disorder. In: Adam MP , Ardinger HH , Pagon RA , Wallace SE , Bean LJ , Stephens K , editors. GeneReviews^®^ [Internet]. University of Washington, Seattle: Seattle (WA); 2020. p. 1993–2020. 33237689

[4] Dupré M , Hermann R , Froment Tilikete C . Update on cerebellar ataxia with neuropathy and bilateral vestibular areflexia syndrome (CANVAS). Cerebellum. 2021;20(5):687–700. 10.1007/s12311-020-01192-w 33011895 PMC8629873

[5] Szmulewicz DJ , McLean CA , Rodriguez ML , Chancellor AM , Mossman S , Lamont D , et al. Dorsal root ganglionopathy is responsible for the sensory impairment in CANVAS. Neurology. 2014;82(16):1410–5. 10.1212/WNL.0000000000000352 24682971 PMC4001192

[6] Montine TJ , Phelps CH , Beach TG , Bigio EH , Cairns NJ , Dickson DW , et al.; National Institute on Aging; Alzheimer’s Association . National Institute on Aging‐Alzheimer's Association guidelines for the neuropathologic assessment of Alzheimer's disease: a practical approach. Acta Neuropathol. 2012;123(1):1–11. 10.1007/s00401-011-0910-3 22101365 PMC3268003

[7] Szmulewicz D , Roberts L , McLean C , MacDougall H , Halmagyi G , Storey E . Proposed diagnostic criteria for cerebellar ataxia with neuropathy and vestibular areflexia syndrome (CANVAS). Neurol Clin Pract. 2016;6(1):61–8. 26918204 10.1212/CPJ.0000000000000215 PMC4753833

